# Identification of novel immune-related targets mediating disease progression in acute pancreatitis

**DOI:** 10.3389/fcimb.2022.1052466

**Published:** 2022-12-14

**Authors:** Qiang Liu, Lingyun Li, Dongchao Xu, Jianpeng Zhu, Zhicheng Huang, Jianfeng Yang, Sile Cheng, Ye Gu, Liyun Zheng, Xiaofeng Zhang, Hongzhang Shen

**Affiliations:** ^1^ Department of Gastroenterology, Affiliated Hangzhou First People’s Hospital, Zhejiang University School of Medicine, Hangzhou, China; ^2^ Department of Gastroenterology, Hangzhou Hospital and Institute of Digestive Diseases, Hangzhou, Zhejiang, China; ^3^ Key Laboratory of Integrated Traditional Chinese and Western Medicine for Biliary and Pancreatic Diseases of Zhejiang Province, Hangzhou, China

**Keywords:** acute pancreatitis, CARS, SIRS, neutrophil, Tlr2-deficient mice

## Abstract

**Introduction:**

Acute pancreatitis (AP) is an inflammatory disease with very poor outcomes. However, the order of induction and coordinated interactions of systemic inflammatory response syndrome (SIRS) and compensatory anti-inflammatory response syndrome (CARS) and the potential mechanisms in AP are still unclear.

**Methods:**

An integrative analysis was performed based on transcripts of blood from patients with different severity levels of AP (GSE194331), as well as impaired lung (GSE151572), liver (GSE151927) and pancreas (GSE65146) samples from an AP experimental model to identify inflammatory signals and immune response-associated susceptibility genes. An AP animal model was established in wild-type (WT) mice and Tlr2-deficient mice by repeated intraperitoneal injection of cerulein. Serum lipase and amylase, pancreas impairment and neutrophil infiltration were evaluated to assess the effects of *Tlr2 in vivo*.

**Results:**

The numbers of anti-inflammatory response-related cells, such as M2 macrophages (*P* = 3.2 × 10^–3^), were increased with worsening AP progression, while the numbers of pro-inflammatory response-related cells, such as neutrophils (*P* = 3.0 × 10^–8^), also increased. Then, 10 immune-related AP susceptibility genes (SOSC3, ITGAM, CAMP, FPR1, IL1R1, TLR2, S100A8/9, HK3 and MMP9) were identified. Finally, compared with WT mice, Tlr2-deficient mice exhibited not only significantly reduced serum lipase and amylase levels after cerulein induction but also alleviated pancreatic inflammation and neutrophil accumulation.

**Discussion:**

In summary, we discovered SIRS and CARS were stimulated in parallel, not activated consecutively. In addition, among the novel susceptibility genes, *TLR2*might be a novel therapeutic target that mediates dysregulation of inflammatory responses during AP progression.

## Introduction

AP is the most common digestive disease and is characterized by inflammation and autodigestion of the pancreas (Vege et al., 2018). The incidence rate of AP is estimated to be 100 to 140 per 100,000 per person annually in developed countries (Mederos et al., 2021). Mild AP (MAP) is a self-limiting disorder that can be resolved within one week with finite treatment (Lee and Papachristou, 2019). However, approximately 10-20% of patients experience moderately severe AP (M-SAP) or severe AP (SAP), which is associated with pancreatic or peripancreatic tissue necrosis, local or systemic complications and persistent single or multiple organ failure and has a high mortality rate (Schepers et al., 2019; Boxhoorn et al., 2020; Mederos et al., 2021). Convincing evidence has demonstrated that infected necrosis is the greatest risk factor for severe AP and mortality, and the intensity of the immune response during this process exerts an enormous influence on systemic complications and disease severity (van Dijk et al., 2017; Sendler et al., 2020). AP is a complex inflammatory disease with diverse characteristics in terms of severity and course. While the therapeutic strategy and stratification systems currently used for AP are generally useful, the effectiveness and accuracy of these methods still need to be improved to reduce the mortality rate and improve early diagnosis of AP (Gukovskaya et al., 2017; Lee and Papachristou, 2019; Leppaniemi et al., 2019; Sun et al., 2021).

AP is triggered by premature activation of digestive enzymes in acinar cells, and the infiltration of inflammatory cells is induced simultaneously (Kono and Rock, 2008; Sendler et al., 2018). In particular, during these processes, immune-related cells undergo interactions under physiological and pathophysiological conditions. Macrophages and neutrophils initially reach the organ and cause pancreatic damage, monocyte induction is a key factor in systemic inflammation and worsening tissue injury, and T-cell activation plays an important role in inducing the adaptive immune response in AP (Gukovskaya et al., 2002; Zheng et al., 2013; Sendler et al., 2018; Xu et al., 2020). As a result of the initial immune response, pro-inflammatory cytokines are released, causing systemic inflammatory response syndrome (SIRS). Meanwhile, hyperinflammation is accompanied by compensatory anti-inflammatory response syndrome (CARS) during disease development (Zheng et al., 2013; Sendler et al., 2020). Abundant hyperinflammation during SIRS is associated with organ dysfunction syndrome or shock in patients, and inordinate immunosuppression during CARS is associated with bacterial translocation, resulting in pancreatic necrosis or severe sepsis; all of these factors dramatically increase the mortality rate of SAP patients (Bhatia and Moochhala, 2004; Lee and Papachristou, 2019). Therefore, elucidating the magnitude and order of induction of SIRS and CARS during different phases of the disease might provide an effective therapeutic strategy for AP.

As pattern-recognition receptors, Toll-like receptors (TLRs) seem to play an essential role in the development and severity of inflammatory diseases by mediating SIRS, regulating inflammatory cell recruitment, altering microvascular leakage and inducing cellular apoptosis (Iwasaki and Medzhitov, 2004; Vaz et al., 2013). Damage-associated molecular patterns (DAMPs) released in cellular contents exert their effects by specifically binding TLRs to activate the NF-kappa B signaling pathway. The induction of NF-kappa B signaling regulates the expression profiles of pro-inflammatory cytokines, chemokines and adhesion factors. Among the 13 subunits of the TLR family, most endogenous ligands and bacteria-derived compounds interact with *TLR2* and *TLR4* to induce the innate immune response (Gorskii et al., 2014; Gorsky et al., 2015). In previous studies, the main candidate gene investigated for targeting in AP was *TLR4*, while the potential functions of *TLR2* in the pathophysiology of AP are still elusive (Vaz et al., 2013). *TLR2* was overexpressed in the peripheral blood, glands and pancreas in a cerulein-induced experimental AP model and in human peripheral blood (Ding et al., 2013; Gorskii et al., 2014). In contrast, Awla and colleagues induced AP in wild-type, *Tlr2-*deficient and *Tlr4*-deficient mice and showed that *Tlr4* but not *Tlr2* regulates chemokine formation, neutrophil recruitment and tissue damage in the SAP mouse model (Awla et al., 2011). Hence, additional *in vivo* and *in vitro* studies are essential for elucidating the effects and biological mechanisms of *TLR2* in the pathogenesis and deterioration of AP.

In this study, we investigated the development and progression of AP on the basis of transcriptional profiles in human peripheral blood, as well as the corresponding signatures in tissue samples from an AP animal model. Consistent with a recent study based on immune cell infiltration analysis and cytokine and chemokine expression patterns in patients with AP of different severities, we found that pro-inflammatory and anti-inflammatory responses were activated in parallel (Sendler et al., 2020), not consecutively, as previously reported for SIRS and CARS (Andersson et al., 2007). Furthermore, to make further progress in the diagnosis and treatment of AP, it is crucial to identify the etiological mechanisms and susceptibility genes of AP *via* comprehensive bioinformatics analysis. Ten immune-related hub genes and pathways for AP were identified based on an integrative analysis. In addition, to investigate the potential functions and biological mechanisms of these immune-related hub genes, AP mouse models were established in WT mice and *Tlr2*-deficient mice. *Tlr2*-deficient mice showed substantial amelioration of the pancreatic inflammatory response and pancreatic injury, with reduced activation of neutrophils. Therefore, these findings highlight that SIRS and CARS are activated simultaneously and that *Tlr2* and several interacting genes that regulate immune cell infiltration during AP progression are potential therapeutic targets for AP.

## Materials and methods

### Data and resources

The count-based gene expression profiles of peripheral blood and associated clinical information for the three AP severity levels were acquired from the Gene Expression Omnibus (GEO) database (accession number: GSE194331), including 32 healthy donors, 57 mild acute pancreatitis (MAP) patients, 20 moderately severe acute pancreatitis (M-SAP) patients and 10 severe acute pancreatitis (SAP) patients (https://www.ncbi.nlm.nih.gov/geo/query/acc.cgi?acc=GSE194331). This dataset was deposited by Maryam N et al. from the Nepean Hospital (Nesvaderani et al., 2022). The severity levels of AP were evaluated according to the Revised Atlanta classification.

To validate the potential targets and etiology of AP in human blood samples, the transcriptome profiles of the AP experimental models GSE151572 (https://www.ncbi.nlm.nih.gov/geo/query/acc.cgi?acc=GSE151572) and GSE151927 (https://www.ncbi.nlm.nih.gov/geo/query/acc.cgi?acc=GSE151927) were downloaded from the GEO database. GSE151572 was designed to gain insight into the effect of emodin in Sprague–Dawley (SD) rats (8 weeks old) with SAP-induced lung injury (Xu et al., 2021a). Emodin- and dexamethasone-treated groups were filtered out, and we acquired the mRNA high-sequencing profiles of only the control group (*n* = 6), SAP-6 h group (*n* = 3) and SAP-24 h group (*n* = 3) to perform further analyses. The SAP model was induced by standard retrograde infusion of fresh 5.0% sodium taurocholate (0.1 mL/100 g body weight) into the biliopancreatic duct. An equal volume of sterile saline was injected into the rats in the control group. Necrosis, inflammation, hemorrhage and edema were more severe in the SAP-24 h group than in the SAP-6 h group according to previous research (Xu et al., 2021a). GSE151927 was designed to extend the understanding of metabolic gene changes during AP based on hepatic transcriptome profiles (Zhang et al., 2022a). The mice were divided into control (*n* = 8), AP (*n* = 8) and SAP (*n* = 8) groups.

Furthermore, to dynamically detect the fluctuating expression levels of causal genes with disease progression, the mRNA profile of pancreas tissue from the AP mouse model in GSE65146 was downloaded (https://www.ncbi.nlm.nih.gov/geo/query/acc.cgi?acc=gse65146). KrasG12D-mutated mice were not included in the subsequent analyses. Hence, data from 44 wild-type mice with cerulein injection-induced AP at 13 consecutive time points remained for further analyses. The expression profiles were evaluated by Affymetrix GeneChip Mouse Gene 1.0 ST arrays.

### Principal component analysis

The count-based gene expression matrix of human and rat models was used to generate the PCA plot with the R packages gmodels (v. 2.18.1) and ggplot2 (v. 3.3.5). Because the 6-hour and 24-hour control groups for the rat model did not receive other treatments, we combined the rats from the two subtypes into one group.

### Differential gene expression analysis

Differentially expressed genes (DEGs) between each severity level of AP (MAP, M-SAP, SAP) and one common healthy group were identified using the DESeq2 package (v. 3.6.3) (Anders and Huber, 2010) in R to detect gene expression changes related to AP aggravation. The genes with a false discovery rate (FDR) ≤0.05 and an absolute value of log2fold change ≥1 were considered to be deferentially expressed (Xu et al., 2021b; Nesvaderani et al., 2022). To limit potential false-positive results, nonexpressed or low-count (average counts ≤ 1) transcripts were filtered out for further analyses. Subsequently, the aberrant expression profiles from different comparison groups in the SAP rat model were analyzed with the same methods described above.

### Immune cell infiltration analysis of the mRNA sequencing data

The CIBERSORTx algorithm was used to evaluate the relative abundance of 22 types of infiltrating immune cells according to the mRNA expression profile (Newman et al., 2015; Newman et al., 2019), which was assessed based on the expression levels of specific markers of immune cells. Before calculating the immune cell proportions, the expression count data were normalized to reads per kilobase per million mapped reads (RPKM) format. The 22 immune cell types involved were naive B cells, memory B cells, CD8^+^ T cells, T cells, follicular helper T regulatory cells, gamma delta T cells, CD4^+^ memory resting T cells, CD4^+^ memory activated T cells, CD4^+^ naive memory T cells, plasma cells, resting natural killer cells, activated natural killer cells, monocytes, M0 macrophages, M1 macrophages, M2 macrophages, resting mast cells, activated mast cells, resting dendritic cells, activated dendritic cells, eosinophils, and neutrophils. Heatmaps were generated and DEG expression profile clustering was performed with gplot (v 3.1.1) and pheatmap (v. 1.0.12) in R.

### Pathway and network analysis

To illuminate the potential mechanisms of AP, the R package ClusterProfiler (v. 3.14.3) was used to perform Kyoto Encyclopedia of Genes and Genomes (KEGG) pathway enrichment analysis (v. 3.14.3) (Yu et al., 2012). Terms with FDR values less than 0.05 were considered to represent significantly enriched pathways. To construct biomolecular networks and identify the causal genes for AP of different severity levels, protein−protein interaction network analysis with overlapping targets was performed using STRING (v. 10.5) with the default parameters (https://string-db.org) (Szklarczyk et al., 2021). Cytoscape (v. 3.9.1) was applied to visualize the biomolecular interactions of SAP-related candidate genes (Shannon et al., 2003). According to the interaction scores, which were calculated with the MCC algorithm scores from the Cytohubba plugin in Cytoscape, the top 10 genes were named hub targets for AP occurrence and severity risk.

The R package WGCNA was implemented to construct the network modules of highly correlated mRNAs (Langfelder and Horvath, 2008). This tool helps to find gene pairs with similar expression patterns and high topological overlap, and it is essential to identify causal genes and understand the etiology of complex diseases. First, using a threshold power of 30, we constructed a weighted network according to the gene pair correlations among all the transcriptomes. Second, 6 specific modules with module sizes from 41 to 10138 were hierarchically clustered using the default parameters to evaluate the network interconnection. In addition, we calculated the correlations among the hub genes’ transcriptome expression profiles using the Spearman method with human and experiential models.

### Mice

C57BL/6J mice were purchased from Charles River Laboratories (Beijing, China). C57BL/6-Tlr2^em1Smoc^ (*Tlr2* ^−/−^) mice were purchased from Shanghai Model Organisms Center, Inc. (Shanghai, China). Only male mice (6-8 weeks) were included in the studies. All mice were housed under conditions of controlled temperature (22-25°C) and humidity (40-60%) with a 12:12 hour light-dark cycle and were allowed free access to food and water. In addition, mice were kept in specific pathogen-free facilities, and all of the experiments were performed in accordance with the guidelines of the Zhejiang University Animal Care and Use Committee.

### Experiential models and treatments

Age-matched wild-type and *Tlr2* ^−/−^ male mice were used in the following experiments. GraphPad software was used to randomize mice with a single sequence of random assignments before treatment. AP was induced by intraperitoneal injection of caerulein (50 μg/kg; Glpbio, Montclair, USA) 8 times at hourly intervals (Sendler et al., 2018; Xu et al., 2020). SAP was induced by hourly injection of caerulein (50 μg/kg) 8 times plus LPS (10mg/kg; Servicebio, Wuhan, China), and LPS was injected right after the last injection of caerulein. Control groups were administered an equivalent volume of phosphate-buffered saline (PBS). Then, the mice were sacrificed 4 hours, 12 hours, 24 hours and 36 hours after the last injection of caerulein. Mice that exhibited signs of suffering during the treatment process were excluded from the study.

### Amylase and lipase measurement

Serum samples were collected from different groups of mice. The amylase and lipase levels were detected with a Beckman Coulter AU680 automatic biochemical analyzer. The remaining blood samples were stored at -80°C until use.

### Histological examination

Hematoxylin and eosin (H&E) staining was applied to detect pancreatic and pulmonary tissue injury in mice with AP or SAP. Paraffin-embedded sections (5 μm) were stained with H&E. For histological examination, the pancreas and lungs were removed and fixed in 4% paraformaldehyde. To identify the pancreas and lungs injury level, multiple randomly selected microscopic fields from at least three mice per group were assessed by two pathologists in a blinded manner. The pancreatic injury score was utilized on the basis of pancreatic edema (0-2), inflammatory cell infiltrate (0-3), hemorrhage and fat necrosis (0-3) and acinar necrosis (0-3), as previously described (Miao et al., 2019). The pulmonary injury score was assessed using a scale for the interstitial and intra-alveolar edema (0-5), interstitial and intra-alveolar leukocyte infiltration (0-5), and fibrosis (0-5), as previously described (Hu et al., 2022).

### Immunohistochemical analysis

Paraffin-embedded sections (5 μm) of pancreatic tissue were incubated with a rabbit polyclonal anti-Ly6G antibody (1:500, Servicebio, Wuhan, China) at 4°C overnight. Further experimental steps were performed according to the manufacturer’s instructions for the Streptavidin/Peroxidase Histostain™ Plus Kit (ZSGQ-BIO, Beijing, China). The number of neutrophils was determined by counting Ly6G^+^ cells per microscopic field (200×).

### Statistical analysis

Statistical analyses and the creation of graphs were performed using the R package Tableone (v. 0.13.0) and GraphPad Prism 8 software. Statistically significant differences were calculated by an unpaired two-tailed Student’s *t test* (2 groups) and one-way ANOVA (multiple groups). A *P* value less than 0.05 was considered to be statistically significant. The correlation matrix was constructed with R according to the Pearson correlation coefficient.

## Results

### DEG analysis and sample clustering for human AP

**Figure 1:**
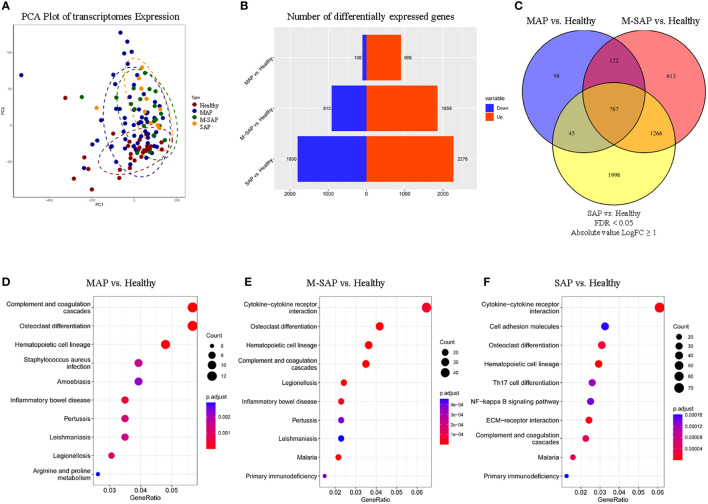
Shared DEGs and pathways of AP of different severity levels. **(A)** Principal component analysis of sub-subjects with MAP, M-SAP or SAP and healthy controls. **(B)** Distribution of the number of DEGs between each severity level of AP and healthy controls. The red rectangle indicates upregulated genes, and the blue rectangle indicates downregulated genes. **(C)** Venn diagram depicting the overlapping DEGs in each severity group compared with a common set of healthy groups. **(D–F)** Pathway enrichment analysis for three severity levels of AP and healthy controls using KEGG analysis. Only the top 10 significantly enriched pathways after sorting by the Bonferonni adjusted *P* value were selected. Dots represent significant pathways, and all of them were sorted and colored by adjusted *P* value.

The mRNA high-throughput sequencing data were collected using blood samples from 32 healthy individuals, 57 MAP patients, 20 M-SAP patients and 10 SAP patients. After quality control, approximately 20,000 annotated transcriptomes were identified in each individual. A PCA plot generated using the gmodels (v. 2.18.1) and ggplot2 (v. 3.3.5) revealed that the characteristic expression profiles of SAP and M-SAP were largely discriminated from those of healthy donors and MAP patients (**Figure 1A**). In contrast, nearly half of the MAP patients were mixed with the healthy donors.

To better understand the molecular mechanisms underlying disease occurrence and risk severity, we conducted DEG analysis with transcriptome sequencing data. When compared directly with that of healthy controls, the number of DEGs dramatically increased with the disease severity, with MAP, M-SAP and SAP exhibiting 906, 1,858 and 2,276 upregulated DEGs and 106, 910, and 1,800 downregulated DEGs, respectively (*P_adj_* ≤ 0.05, |log2FC| ≥ 1; **Figure 1B**; **Tables S1-3**). In addition, there were 934 significant DEGs (92.9%) for MAP that were shared between M-SAP or SAP. Of these genes, 767 DEGs, which accounted for more than three-fourth of the MAP DEGs (75.8%), were abnormally expressed in a consistent direction in both M-SAP and SAP (27.7%, 18.8%; **Figure 1C**). These results suggested that the majority of DEGs for MAP also play essential roles in progression to M-SAP and SAP.

### DEGs-based enrichment analysis for three severity levels of AP

To illuminate the potential pathogenesis and shared mechanisms of different severity levels of AP, the DEGs were subjected to KEGG enrichment analysis. Our pathway analysis of DEGs for different severity levels of AP (MAP, M-SAP, and SAP) compared against one common control group revealed 8, 13 and 26 significantly enriched pathways (FDR ≤ 0.05), respectively (**Table S4**; **Figures 1D-F**). Of these pathways, six of the eight pathways enriched in MAP (complement and coagulation cascades, osteoclast differentiation, hematopoietic cell lineage, inflammatory bowel disease, legionellosis and pertussis) were also significantly enriched in M-SAP and SAP. For MAP, the most significantly enriched pathway was the complement and coagulation cascades (*P* = 6.02 × 10^–7^); this pathway was significantly enriched in the M-SAP and AP groups (*P* = 1.65 × 10^–7^ *, P* = 2.74 × 10^–3^). Interestingly, a previous study demonstrated that this pathway was significantly activated in acute necrotizing pancreatitis patients compared with interstitial edema pancreatitis patients, and crucial genes in this pathway were positively related to pancreatic necrosis and aggravated AP (Zhang et al., 2022b). The inflammatory bowel disease pathway was a common pathway in the three groups (*P* = 4.39 × 10^–4^ *, P* = 4.94 × 10^–5^ *, P* = 6.54 × 10^–3^), confirming an important role of this pathway in AP. According to the clinical presentation, pancreatic abnormalities are common in inflammatory bowel disease (IBD) patients, and nearly 20% of IBD patients have asymptomatic exocrine insufficiency and/or hyperamylasemia (Ramos et al., 2016). The wide spectrum of pancreatitis-related symptoms observed in IBD may reveal the shared etiology and shared common genes of the two diseases.

According to the pathway analysis, gradually aggravated immune dysfunction was identified. We identified 13 significantly dysregulated pathways in M-SAP, with *P* values ranging from 1.65 × 10^–8^ to 1.96 × 10^–3^. Notably, 12 dysregulated pathways in MAP were significantly enriched in SAP, such as hematopoietic cell lineage and cytokine−cytokine receptor interaction (M-SAP: *P* = 1.27 × 10^–7^ *, P* = 4.72 × 10^–5^; SAP: *P* = 7.51 × 10^–5^ *, P* = 4.25 × 10^–4^). For SAP, 26 dysregulated pathways were detected, with *P* values ranging from 7.52 × 10^–5^ to 4.51 × 10^–2^. In addition, the majority of these pathways were highly related to immune responses, such as Th17-cell differentiation and the NF-kappa B signaling pathway (*P* = 5.00 × 10^–3^ *, P* = 5.00 × 10^–3^), which indicated a stronger perturbation of inflammation in SAP.

### Distinct characteristics of immune infiltration in AP

**Figure 2:**
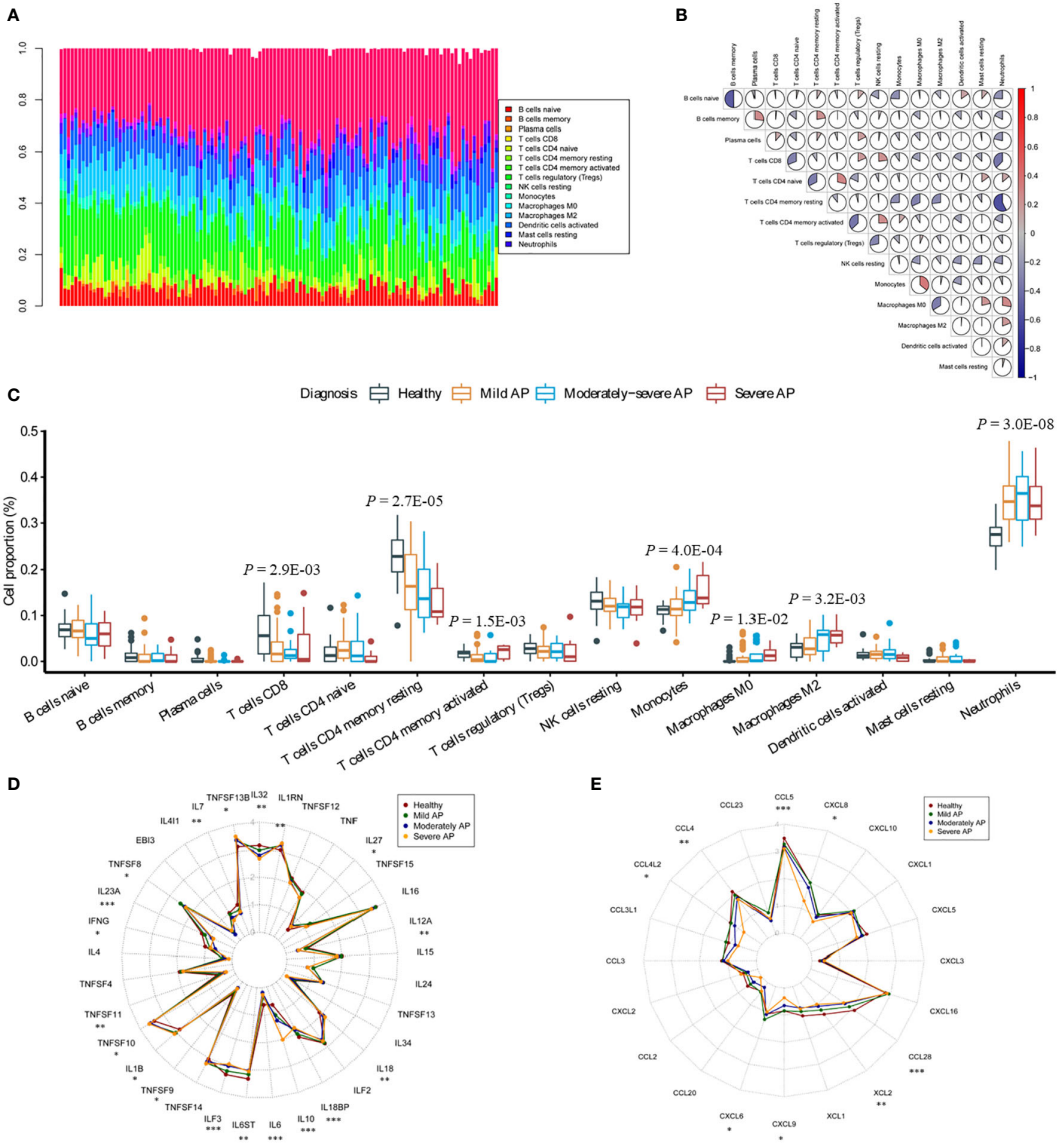
Analysis of immune cell infiltration and the transcription profile of cytokines and chemokines in AP. **(A)** The proportions of 22 immune-related cells were assessed through the CIBERSORTx algorithm in AP. The stacking plot indicates the distribution of 22 types of immune cells in AP. **(B)** The correlation matrix of 22 types of immune cells in AP. **(C)** Significant differences among the groups were evaluated utilizing ANOVA. The Y-axis indicates the percentage of immune cells in AP. **(D, E)** Dot plots represent the expression levels of cytokines and chemokines in the peripheral blood of humans. Asterisks indicate significant differences, red represents healthy donors, dark green represents MAP patients, dark blue represents M-SAP patients, and dark yellow represents SAP patients (*P < 0.05; **P < 0.01; ***P < 0.001).

Using CIBERSORT, we presented the landscape of 22 infiltrating immune-related cell populations in AP by analyzing the mRNA expression profile of the GSE194331 dataset (**Figure 2A**). Among the 22 types of immune cells, follicular helper T cells, gamma delta T cells, activated NK cells, M1 macrophages, activated mast cells, resting dendritic cells, and eosinophils were not detected based on the peripheral blood RNA-seq dataset (**Table S5**). We also illustrated the relationships between different types of immune-related cells in AP using a correlation matrix. Interestingly, neutrophils exhibited negative correlations with naive B cells, memory B cells, CD8^+^ T cells, resting memory CD4^+^ T cells, activated memory CD4^+^ T cells, and resting natural killer cells and positive relationships with M0 and M2 macrophages (**Figure 2B**). The proportion of monocytes demonstrated a positive correlation with M0 macrophages and a negative correlation with naive B cells and resting memory CD4^+^ T cells. Furthermore, we investigated the differences in these immune-related cell proportions among AP of three severity levels and healthy controls. Using ANOVA, the fractions of CD8^+^ T cells and resting memory CD4^+^ T cells were clearly decreased with increasing AP severity (*P* = 2.9 × 10^–3^ *, P* = 2.7 × 10^–5^). The proportions of activated memory CD4^+^ T cells, monocytes, M0 macrophages, M2 macrophages and neutrophils were significantly increased among the four groups (*P* = 1.5 × 10^–3^ *, P* = 4.0 × 10^–4^, *P* = 1.3 × 10^–2^ *, P* = 3.2 × 10^–3^ and *P* = 3.0 × 10^–8^, respectively; **Figure 2C**).

To investigate the immune response in more detail, we evaluated the whole-blood cytokine and chemokine mRNA expression profiles of different severity levels of AP and the healthy group (**Table S6**). During disease progression, cytokines and chemokines are released due to acinar cell injury and necrosis; these factors recruit and mediate the infiltration of immune cells into the injury area (Lee and Papachristou, 2019). Blood cytokine profiles revealed that the pro-inflammatory response indicators *TNFSF13β, IL1β* and *IL18* were significantly elevated with the aggravation of AP (*P* = 0.010, *P* = 0.031, *P* = 0.004, respectively; **Figure 2D**). In contrast, compared with those in the healthy groups, the transcript levels of several pro-inflammatory cytokines, such as the *IL12* cytokine family *IL12α* and *IL23α*, *IL6, IL32* and *IFN-γ* (*P* = 0.007, *P* < 0.001, *P* < 0.001, *P* < 0.001, *P* = 0.001, respectively) were markedly decreased. Furthermore, expression of the anti-inflammatory cytokine *IL10* (*P* < 0.001) was significantly elevated. In addition, compared with that in the healthy group, expression of *CCL4, CCL4L2, CCL5, CCL28, XCL2, CXCL6, CXCL8* and *CXCL9* was significantly decreased (*P* = 0.003, *P* = 0.025, *P* < 0.001, *P* < 0.001, *P* = 0.003, *P* = 0.021, *P* = 0.029, *P* = 0.040, respectively; **Figure 2E**).

### Immune-related genes identified in humans and experimental AP models

To further understand the pathogenesis of AP, we searched for DEGs for the common control groups *vs.* the SAP-6 h groups and the common control groups *vs.* the SAP-24 h groups. According to the PCA plot, the characteristics of the control and SAP-6 h groups were dramatically different from those of the SAP-24 h group (**Figure S1**). A total of 20,342 high-quality rat transcripts remained after quality control. Compared with the control group and SAP-6 h group, the SAP-6 h group exhibited 123 significantly downregulated transcripts and 167 upregulated transcripts (*P_adj_* ≤ 0.05, |log2FC| ≥ 1; **Figure S2a**; **Table S7**). Next, compared with a common set of control groups and the SAP-24 h groups, 471 transcripts were significantly downregulated, and 652 transcripts were upregulated (**Figure S2b**; **Table S8**).

Furthermore, to comprehensively understand the etiology and mechanism of different severity levels of AP, the abovementioned DEGs were subjected to pathway enrichment analysis. In the KEGG analysis results for the control group *vs.* SAP-6 h group, we observed 9 significantly enriched KEGG terms with *P* values ranging from 1.71 × 10^–5^ to 2.27 × 10^–3^ (**Figure S2c** and **Table S9**). In addition, for the control group *vs.* SAP-24 h group, the number of enriched pathways was dramatically increased, and the majority of these pathways were strongly related to dysfunctional immune responses (**Figure S2d**). We identified 28 significant pathways with *P* values ranging from 6.19 × 10^–15^ to 1.72 × 10^–3^. Four pathways, namely, hematopoietic cell lineage, complement and coagulation cascades, ECM-receptor interaction and proteoglycans in cancer, were identified as common pathways in the rat experimental AP model. Notably, the hematopoietic cell lineage and complement and coagulation cascade pathways were identified as common pathways in both the human and experimental AP models.

**Figure 3:**
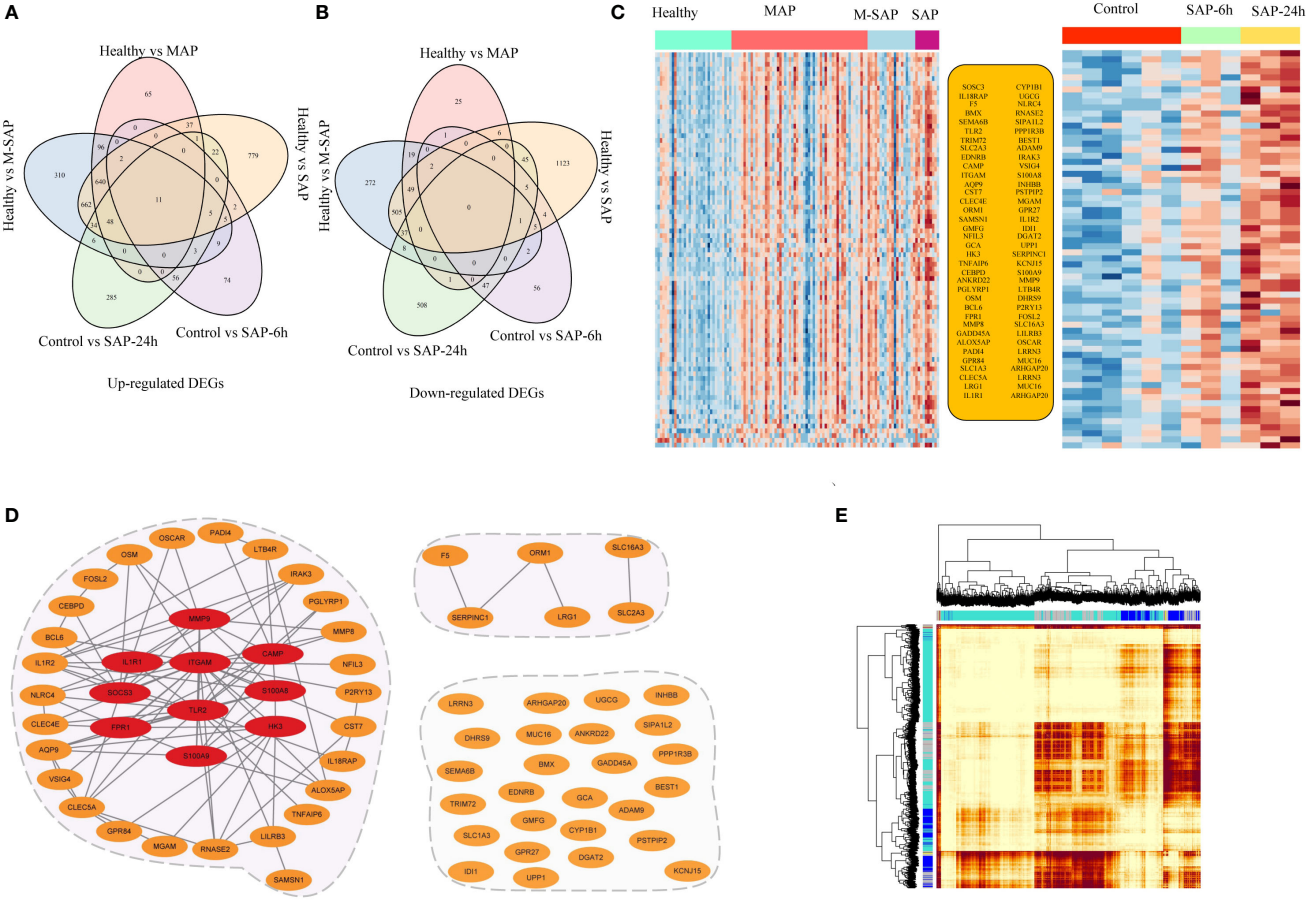
Specific transcriptional characteristics and potential interaction mechanisms of AP-associated genes. **(A, B)** The Venn diagram represents the overlapping genes from human blood samples and the animal AP model. **(C)** Expression of 71 shared transcriptomes according to DEG analysis of human blood samples and samples from the animal AP model. **(D)** Interaction network analysis was applied using the STRING database and WGCNA. Orange dots represent the 71 AP-related genes. The red ellipse denotes the 10 hub genes that were scored with the cytoHubba tool. The gray edge represents the interaction relationships annotated by the STRING database. **(E)** Topological overlap matrix plots for AP modules.

Based on the transcriptional profiles of humans and experimental rat models, there were 69 upregulated DEGs and 3 downregulated DEGs for AP that were shared among at least four different groups (**Figures 3A-C**). Next, protein−protein interaction network (PPI) analysis was conducted to identify the interactions among the overlapping genes using the STRING database (https://string-db.org/cgi/input.pl) with the default parameters (**Figure 3D**). The results showed that 43 genes strongly interacted with each other. The R package of WGCNA was applied to identify the gene pairs with highly topological overlap and strongly related expression patterns, and it is important to detecting promising targets genes and understanding the pathology of diseases. Performing with the default parameter, 6 specific modules were hierarchically clustered (**Figure 3E**). According to the weighted network module, among of the 43 highly related genes, 35 genes with similar expression profiles and located in blue module (*n* = 3,037; **Figure S2e**). Furthermore, the top ten highly interacting hub genes were screened based on the interaction score, which was calculated through the 12 different algorithms of Cytohubba software (**Figures S3a-i**). Interestingly, similar to the MCC algorithm analysis result, *TLR2* was annotated as one of top 10 hub genes in Betweenness, BottleNeck, Closeness, Degree, EPC, MNC, Radiality, Stress algorithms analyzed results, except under ClusteringCoefficient, DMNC and EcCentricity model. Therefore, these results indicated that *TLR2* might play important role in the etiology and mechanism of AP.

**Figure 4:**
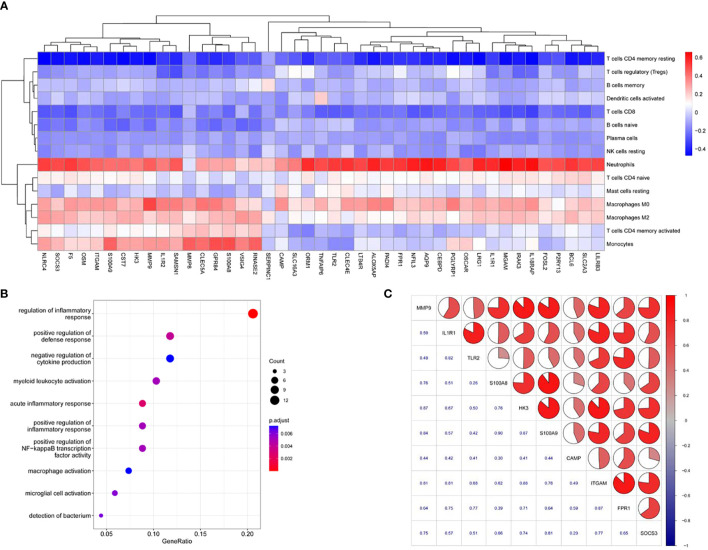
The potential functions of AP-related genes. **(A)** Heatmap denoting the relationships among the identified 43 highly connected candidate genes and immune cell proportions. Colored rectangles represent the Spearman correlation coefficient of transcript abundance for the highly connected candidate genes and fluctuating immune cell proportions. **(B)** GO term enrichment analysis of 43 highly connected candidate genes for AP. **(C)** Correlation matrix of the expression profiles of hub genes of AP in human blood samples.

To infer the potential functions of the hub genes for AP, we first performed correlation analysis between the hub genes and immune-related infiltrating cells in AP. The heatmap revealed that *SOSC3, ITGAM, CAMP, FPR1, IL1R1, TLR2, S100A8/9, HK3* and *MMP9* were negatively correlated with CD8^+^ T cells and resting memory CD4^+^ T cells, and the majority of these genes were positively correlated with activated memory CD4^+^ T cells, monocytes, M0 macrophages, M2 macrophages and neutrophils (**Figure 4A**). Second, since we were unable to annotate the GO terms for some of the 10 hub genes for AP, we performed GO analysis using ClusterProfiler with the 43 highly interacting hub genes of AP to identify overrepresented biological processes, and only terms with FDR values < 0.05 were considered significant. The GO analysis revealed enrichment of biological processes largely related to immune response (e.g., regulation of inflammatory response and macrophage activation; **Figure 4B** and **Table S10**). Interestingly, nine of ten hub genes were included in the gene set of the blue module; only *CAMP* was excluded. In addition, we utilized Spearman correlation analysis to assess the relationships among the 10 hub genes for AP. The hub gene expression levels were positively correlated with each other, with Spearman correlation coefficients ranging from 0.26 to 0.90 in humans and from 0.51 to 0.99 in the experimental AP model (**Figure 4C**; **Figure S2f**). Therefore, it can be inferred that the ten hub genes for AP closely interact and probably exert their effects by regulating the immune response in AP.

### Deletion of TLR2 attenuates the severity of AP

**Figure 5:**
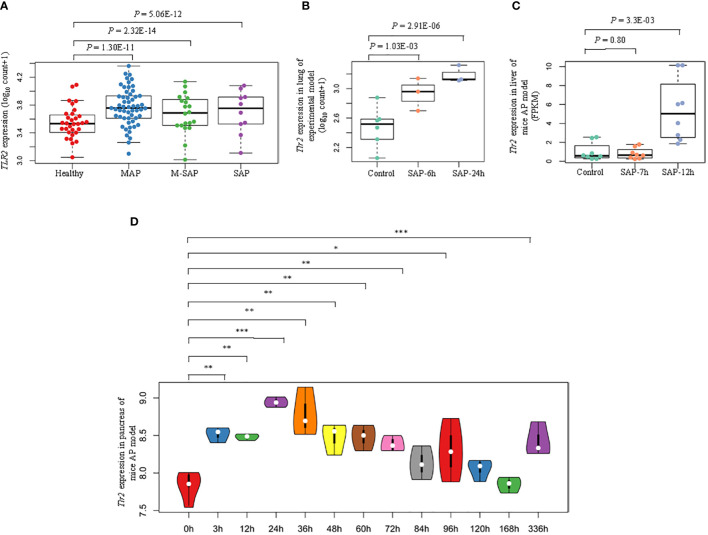
Schematic diagram of identified epigenetic aberrations of *TLR2* in AP. **(A)** Box plots indicate the differential expression patterns of *TLR2* between each severity level of AP (MAP, M-SAP, SAP) and normal controls in human peripheral blood. **(B)** Box plots represent the differential expression profiles of *Tlr2* at 6 hours and 24 hours after lung injury and **(C)** at 7 hours and 12 hours after liver injury in the cerulein-induced AP mouse model. **(D)** Violin plot indicating dynamic fluctuations in *Tlr2* expression at thirteen consecutive time points in pancreatic tissue from the AP mouse model: *P < 0.05; **P < 0.01; ***P < 0.001.

Bioinformatic analyses showed that the hub genes for AP were overexpressed during the induction and progression of AP (**Figures S4a-i**). Compared with those in the healthy group, *TLR2* expression levels in blood samples from MAP, M-SAP and SAP patients were dramatically upregulated (*P* = 4.19 × 10^–10^, *P* = 1.24 × 10^–14^, *P* = 5.19 × 10^–11^; **Figure 5A**). In addition, in accordance with the results obtained for human blood samples, *Tlr2* was gradually increased in injured lung tissue from the caerulein-treated group compared with the control group at 6 hours and 24 hours (*P* = 0.02, *P* = 9.76 × 10^–05^; **Figure 5B**). In addition, the dysregulated expression profile was identified in injured liver tissue from caerulein-treated mice at 7 hours and 12 hours (*P* = 0.80, *P* = 3.3 × 10^–03^; **Figure 5C**). Furthermore, during the recovery phase of AP, compared with 0 hour time point for cerulein-injected mice, the results from AP model mice at 13 consecutive time points indicated that the *Tlr2* expression level in pancreas tissue dramatically increased and peaked at 24 hours and then gradually recovered (0 h *vs.* 3 h: *P* = 1.21 × 10^–3^, 0 h *vs.* 12 h: *P* = 1.20 × 10^–3^, 0 h *vs.* 24 h: *P* = 6.97 × 10^–5^, 0 h *vs.* 36 h: *P* = 1.67 × 10^–3^; 0 h *vs.* 48 h: *P* = 4.04 × 10^–3^, 0 h *vs.* 60 h: *P* = 2.81 × 10^–3^, 0 h *vs.* 72 h: *P* = 4.54 × 10^–3^, 0 h *vs.* 84 h: *P* = 0.093, 0 h *vs.* 96 h: *P* = 0.043, 0 h *vs.* 120 h: *P* = 0.081, 0 h *vs.* 168 h: *P* = 0.952, 0 h *vs.* 336 h: *P* = 6.85 × 10^–3^, **Figure 5D**). Regarding other hub genes, *Itgam, Socs3, Mmp9, Il1r1, Hk3* and *Fpr1* expression levels were significantly increased after caerulein injection and peaked before 12 hours in the mouse model, but *S100a9* and *Camp* did not show the same pattern (**Figures S5a-i**).

**Figure 6:**
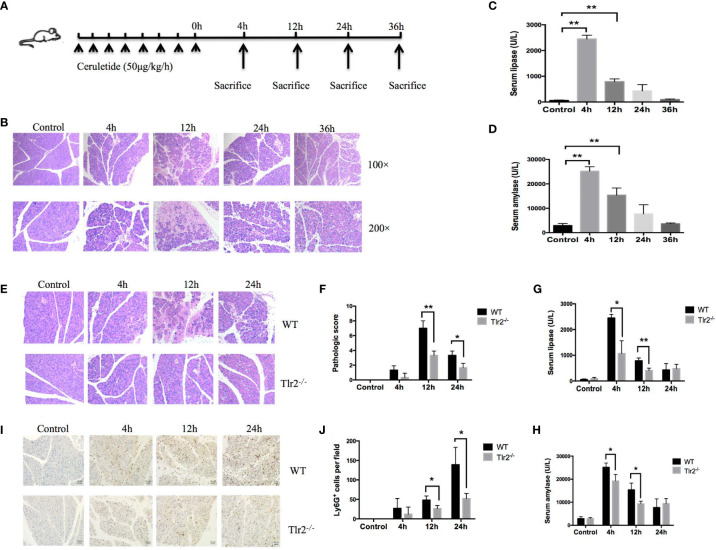
*Tlr2* deficiency alleviated pancreatic inflammation in a cerulein-induced AP mouse model. **(A)** Illustration of the experimental protocol. **(B)** Histological analysis of mouse pancreases in the cerulein-induced acute pancreatitis mouse model (original magnification, 100× or 200×). **(C, D)** Serum lipase and amylase were detected in a cerulein-induced acute pancreatitis mouse model. **(E)** H&E staining of pancreas samples from wild-type (WT) and *Tlr2* ^-/-^ mice (original magnification, 200×). **(F)** Pancreas injury scores were evaluated based on H&E staining. **(G, H)** Serum lipase and amylase levels in the WT and *Tlr2* ^-/-^ groups are displayed. **(I)** Immunostaining for neutrophils was performed. The sections were stained for Ly6G to confirm the infiltration of neutrophils in the pancreas (original magnification, 200×). **(J)** The numbers of Ly6G+ cells were counted based on immunostaining. Data are shown as the mean ± SD (*n* = 3/group). These data are representative of three independent experiments. *P < 0.05; **P < 0.01.

To prove that *Tlr2* is a crucial gene for AP, an animal model of AP was established through repeated injection of cerulein 8 times at hourly intervals in C57BL/6J mice and *Tlr2* ^−/−^ mice (**Figure 6A**). As shown in **Figure 6B, H, E** staining of pancreatic tissues showed that inflammatory infiltration and edema continually increased and peaked at 12 hours as AP developed and then gradually recovered at 36 hours. The serum lipase and serum amylase activity levels were dramatically elevated in AP mice and peaked at 4 hours (**Figures 6C, D**). These data indicated that we successfully established a MAP mouse model with this method, resulting in intrapancreatic necrosis on histology and increased serum lipase and serum amylase compared with control mice. Next, to confirm *Tlr2* as a risk gene that plays an important role in the pathogenesis of AP, we induced AP in *Tlr2-*deficient mice and C57BL/6J mice. H&E staining indicated that *Tlr2^−/−^* mice exhibited a significant alleviation of pancreatic tissue damage compared to that in wild-type mice (**Figures 6E, F**). Furthermore, cerulein treatment resulted in significantly increased serum lipase and amylase activity levels in both WT and *Tlr2* ^−/−^ mice. However, after cerulein induction, the serum lipase and amylase activity levels of *Tlr2* ^−/−^ mice were clearly lower at 4 hours and 12 hours than those of WT mice (**Figures 6G, H**). Next, immunohistochemical staining was performed to examine neutrophil infiltration in the pancreas. Significantly increased neutrophil numbers were observed in the pancreas in wild-type mice at 12 hours and 24 hours. In contrast, neutrophil infiltration in the pancreas was significantly decreased in the *Tlr2^−/−^* groups compared to the wild-type groups (**Figures 6I, J**).

**Figure 7:**
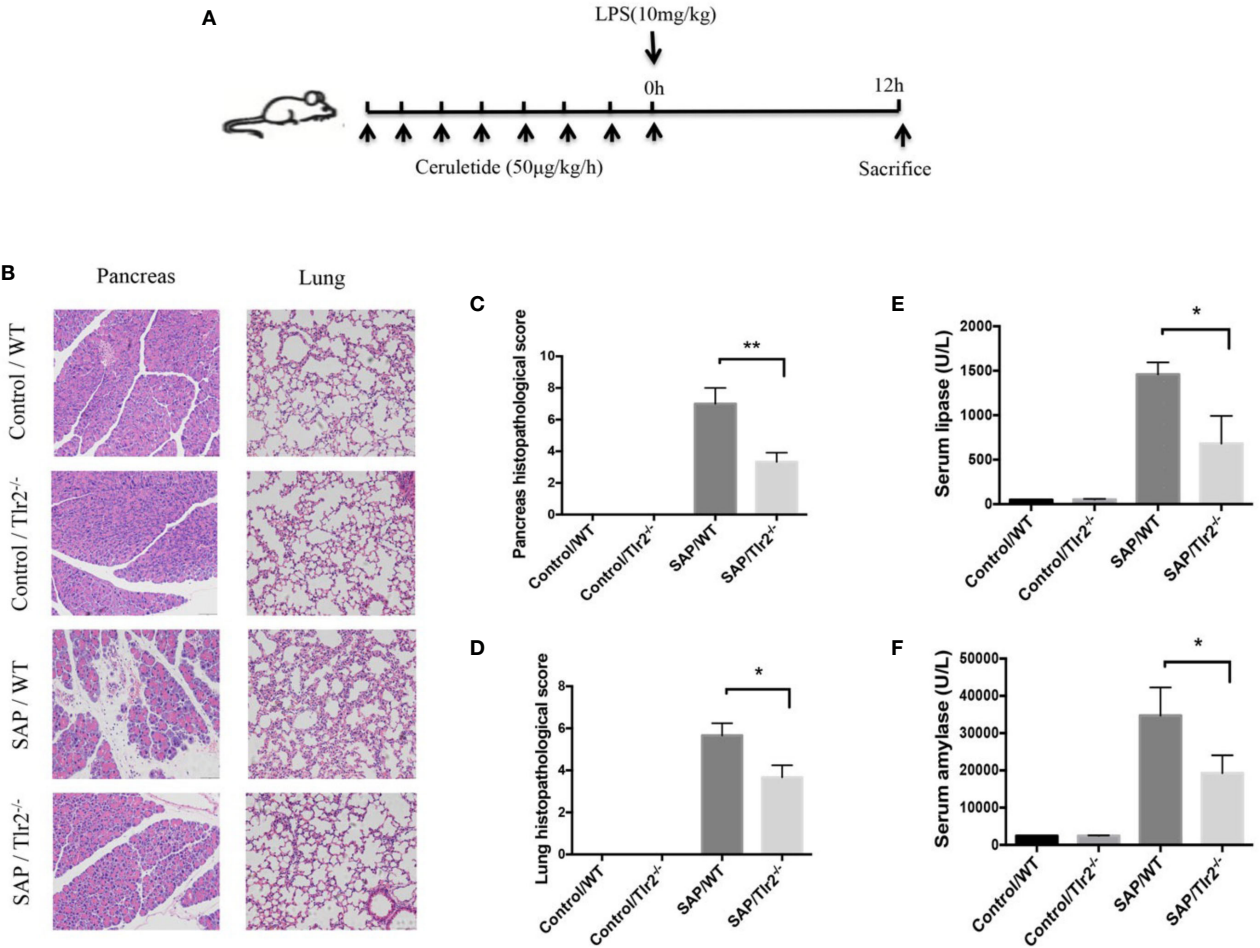
*Tlr2* deficiency alleviated pancreatic and pulmonary injury in a SAP mouse model. **(A)** Illustration of the experimental protocol. **(B)** H&E staining of pancreas and lungs samples from wild-type (WT) and *Tlr2* ^-/-^ mice (original magnification, 200×). **(C)** Pancreatic injury scores were evaluated based on H&E staining. **(D)** Pulmonary injury scores were evaluated based on H&E staining. **(E, F)** Serum lipase and amylase levels in the WT and *Tlr2* ^-/-^ groups are displayed. Data are shown as the mean ± SD (*n* = 3/group). These data are representative of two independent experiments. *P < 0.05; **P < 0.01.

To confirm the effect of *Tlr2* on SAP, an animal model of SAP that mimicking septic conditions with multiple organ failure was established through LPS (10mg/kg) superimposed on a caerulein*8 regimen right after the last injection of caerulein (**Figure 7A**). H&E staining indicated that *Tlr2^−/−^* mice exhibited a significant alleviation of pancreatic tissue damage compared to that in wild-type mice (**Figures 7B, C**). Lung injury is the most common extra pancreatic organ dysfunction induced by SAP. We also investigated the lung injury in the different groups. As shown in (**Figures 7B, D**), Tlr2−/− mice also exhibited a significant alleviation of lung tissue damage compared to that in wild-type mice. What is more, the serum lipase and amylase activity levels of *Tlr2* ^−/−^ mice were lower than those of WT mice (**Figures 7E, F**). Taken together, these results show that overexpression of *TLR2* increased disease severity and pancreatic damage in AP patients and animal models.

## Discussion

AP is an inflammatory disease of the pancreas that can lead to SIRS and CARS, which are associated with a high mortality rate (Gentile et al., 2012; Zerem, 2014; Hawiger, 2018). This study advances two concepts: the order of induction of SIRS and CARS and immune-related susceptibility genes during AP initiation and progression. On the one hand, our results strongly suggested that SIRS and CARS were induced concurrently, not consecutively, during the course and aggravation of AP. On the other hand, 10 immune-related hub genes for AP, namely, *SOSC3, ITGAM, CAMP, FPR1, IL1R1, TLR2, S100A8/9, HK3* and *MMP9*, were identified in patients with different severity levels of AP compared to a common set of healthy donors, and these dysregulated expression levels were validated in various tissues from an AP experimental model. Among the central genes, *S100A8/9, MMP9, SOCS3* and *IL1R1* have been widely reported to be associated with the progression or tissue injuries in AP (Norman et al., 1996; Farkas et al., 2014; Shi et al., 2018; Qin et al., 2019). Therefore, *CAMP, FPR1, TLR2, HK3* and *ITGAM* are novel targets for AP that need to be given more attention in future studies. The *TLR* family plays an essential role in AP by supporting recognition of conserved structures of microorganisms by immune cells and promoting SIRS to induce tissue damage; however, the effects of *TLR2* in AP remain controversial (Awla et al., 2011; Gorskii et al., 2014; Lee and Papachristou, 2019). Hence, we performed several experiments *in vivo* to highlight *TLR2* as a risk gene for AP that amplifies local inflammation and pancreatic injuries by inducing pro-inflammatory neutrophil activation.

Abnormal immune responses are an essential contributor to the pathogenesis of AP (Mayerle et al., 2012; Lee and Papachristou, 2019). Notably, previous studies indicated that AP patients’ disease progression and treatment outcomes are strongly related to innate and adaptive immune cell infiltration (Bhatnagar et al., 2001; Hatano et al., 2001; Van Gassen et al., 2015; Caluianu et al., 2017; Schmidt et al., 2017; Zhao et al., 2018; Yang et al., 2021). However, because of the inability to obtain resected pancreatic tissue from AP patients and the limited number of immune cells obtained from the pancreas in the AP mouse model, a systemic analysis of infiltrating immune cells during the different phases of AP is currently lacking. Hence, we conducted microenvironment analysis with a large number of transcriptome profiles from AP patients’ blood samples by using a transposed convolution algorithm in CIBERSORTx. This analysis provides integrative and dynamic insights into the regulatory network between AP progression and immune cell infiltration. CD4^+^ T cells and CD8^+^ T cells play an essential role in inducing pro-inflammatory cytokine production and provoking the immune response during the course of AP (Mayerle et al., 2012; Xu et al., 2020). In addition, activating CD4^+^ T cells amplified the adaptive immune response and enlarged pathological changes in AP of different severity levels (Sendler et al., 2020; Xu et al., 2020). However, Yang et al. showed that CD4^+^ T cells were significantly downregulated in pancreatic tissue in cerulein-induced or alcohol- and palmitoleic acid-induced MAP mouse models (Yang et al., 2021). In our study, we found that the numbers of CD8^+^ T cells and resting memory CD4^+^ T cells were significantly decreased and that those of activated memory CD4^+^ T cells were significantly increased following disease aggravation. Previous studies indicated that the pro-inflammatory response is activated in the early stage and that the anti-inflammatory response is activated in the later stage of AP, which is characterized by high expression of anti-inflammatory cytokines and anti-inflammatory immune cell infiltration (Lee et al., 2017; Lee and Papachristou, 2019). We found not only anti-inflammatory cells, including M0 macrophages and M2 macrophages, were significantly activated, followed by the worsening of AP progression but also that production of anti-inflammatory related cytokines was significantly stimulated. For example, expression of the anti-inflammatory cytokine *IL10*, which plays an essential role in increasing the Treg response, was significantly elevated during AP aggravation (Sendler et al., 2020). In addition, transcriptional analysis of chemokines and chemokine receptors supported the idea that anti-inflammatory responses and inflammatory responses emerge simultaneously in AP. The significantly dysregulated chemokines and chemokine receptors were all downregulated. However, previous studies reported that inhibition of chemokines and chemokines in an AP mouse model exerted protective effects against pancreas and distant organ injury (Frossard et al., 2011; Malla et al., 2016). These inconsistent results suggest that SIRS and CARS are activated simultaneously during the progression of AP, and balancing the intensity and order of induction of the immune response might be an effective therapeutic approach for AP.

Many studies have shown that the course and severity of AP are largely determined by the crosstalk between innate and adaptive immune responses (Sendler et al., 2020; Xu et al., 2020; Nesvaderani et al., 2022), yet the essential genes remain largely unclear. In the current study, by conducting an integrative analysis of samples from AP patients and injured tissues from an AP animal model, we intended to explore the susceptibility genes and biological pathways involved in the pathogenesis of AP. As a result, we highlighted 43 highly interacting DEGs that were common to at least four different groups. Spearman correlation analysis indicated that the majority of 43 candidate genes were positively correlated with neutrophil, M0 and M2 macrophage cell proportions and negatively correlated with the proportions of adaptive immune cells, such as resting memory CD4^+^ T cells and CD8^+^ T cells. More interestingly, enrichment analysis showed that the 43 susceptibility genes in AP were significantly enriched in biological processes related to the immune response, which is consistent with the notion that function-related genes contribute to the risk of complex disease (Chen et al., 2011). In addition, the MCC algorithm scores from cytoHubba were used to select *SOSC3, ITGAM, CAMP, FPR1, IL1R1, TLR2, S100A8/9, HK3* and *MMP9* as the top 10 enriched genes for AP. Notably, 5 of 10 hub genes have been reported to be associated with physiological and pathophysiological phenotypes of AP. For example, as a calcium sensor, *S100A8/9* forms a heterodimer that is constitutively expressed in neutrophils and monocytes, contributing to the induction of leukocyte recruitment and the stimulation of cytokine secretion during the inflammatory response (Wang et al., 2018). Several studies have shown that *MMP9* is stimulated by *S100A8/9* under inflammatory conditions to strengthen the immune response during the progression of AP (Shi et al., 2018; Wang et al., 2018). In the SAP experimental model, LPS-induced overexpression of *Sosc3* exacerbated MAP, leading to SAP (Zhou et al., 2015). In addition, we detected that a large proportion of the 10 genes were involved in the blue network module based on WGCNA, and the fluctuating expression patterns of these genes showed positive correlation in human and animal models. These results indicate that these targets may perform similar functions to mediate immune cell infiltration and the immune response and influence the initiation and progression of AP.

TLRs perform multiple functions in different types of immune cells, and TLR signaling is related to the activation of adaptive immunity. There are still large gaps in our understanding of TLRs (Fitzgerald and Kagan, 2020). Although *TLR4* has been widely reported to be associated with increased SIRS and tissue injury, the biological functions of *TLR2* in AP remain controversial (Awla et al., 2011; Gorskii et al., 2014; Lee and Papachristou, 2019). Interestingly, based on an integrative analysis, we found that *TLR2* was dramatically overexpressed in blood samples from MAP, M-SAP and SAP patients compared with healthy donors, with similar expression profiles in impaired pancreas and lung tissues from animal models of MAP and SAP. These findings are consistent with a previous study showing that *TLR2* was overexpressed in peripheral blood mononuclear cells from AP patients compared with healthy controls (Gorskii et al., 2014). However, a paradoxical result indicated that a *Tlr2*-deficient mouse model did not exhibit an obvious difference from WT mice after treatment with taurocholate (Awla et al., 2011). These contradictory results might result from the use of different methods to induce MAP. Our study showed that *Tlr2* deficiency in mice with cerulein-induced AP resulted in alleviated pancreas injury and reduced neutrophil accumulation within the pancreas during AP compared with those in WT mice. Herein, our findings imply that *TLR2* was a key factor and was significantly activated in MAP, M-SAP and SAP patients, contributing to the severity of AP, leading to the accumulation of neutrophil infiltration, and increasing pancreatic necrosis and distant organ impairment.

Several limitations of the current study are worth noting. First, although we presented a variety of evidence from human peripheral blood and animal models to support the conclusion that the 10 susceptibility genes of AP are linked to immune cell infiltration, we could not determine to what extent each gene contributes to regulating the immune response in AP, nor could we further assess the regulation of these targets. Second, we speculate that sustaining dysregulated *TLR2* expression would be even worse in clinical patients with SAP or in an aggravated AP animal model with more severe SIRS. Thus, more studies are needed to evaluate the contributions and functions of *TLR2* in SAP patients and SAP mouse models.

In summary, by conducting an integrative bioinformatics analysis of transcriptional sequencing data from human peripheral blood and AP animal models, we showed that pro-inflammatory and anti-inflammatory responses induce the progression of AP in parallel and synergistically. In addition, we discovered 10 highly connected susceptibility genes that regulate the immune response to influence the course and severity of AP. Subsequently, we found that knockout of *Tlr2* alleviated organ impairment and neutrophil accumulation in the pancreas. Therefore, by pinpointing which immune responses and susceptibility genes affect the initiation and aggravation of AP and investigating the underlying mechanisms, this study provides a solid foundation for understanding the progression of AP and identifies several novel therapeutic targets that mediate the dysregulation of pro-inflammatory and anti-inflammatory responses during AP.

## Data availability statement

The datasets presented in this study can be found in online repositories. The names of the repository/repositories and accession number(s) can be found in the article/**Supplementary Material**.

## Ethics statement

The animal study was reviewed and approved by The Zhejiang University Animal Care and Use Committee.

## Author contributions

QL, LL, and DX collected the data, performed the bioinformatic analysis, AP animal model experiments, and wrote the manuscript. JZ, ZH, SC, LZ, and YG were involved in data collection and reviewed the manuscript. XZ and HS conceived the current study and reviewed the manuscript. All authors contributed to the article and approved the submitted version.
