# Supplementary material for: Identification of novel immune-related targets mediating disease progression in acute pancreatitis
Source: Front Cell Infect Microbiol. 2022 Dec 14;12:1052466. doi: 10.3389/fcimb.2022.1052466 (PMC9795030; doi:10.3389/fcimb.2022.1052466)
Supplement: Supplementary file 1 [file DataSheet_1.docx]

**
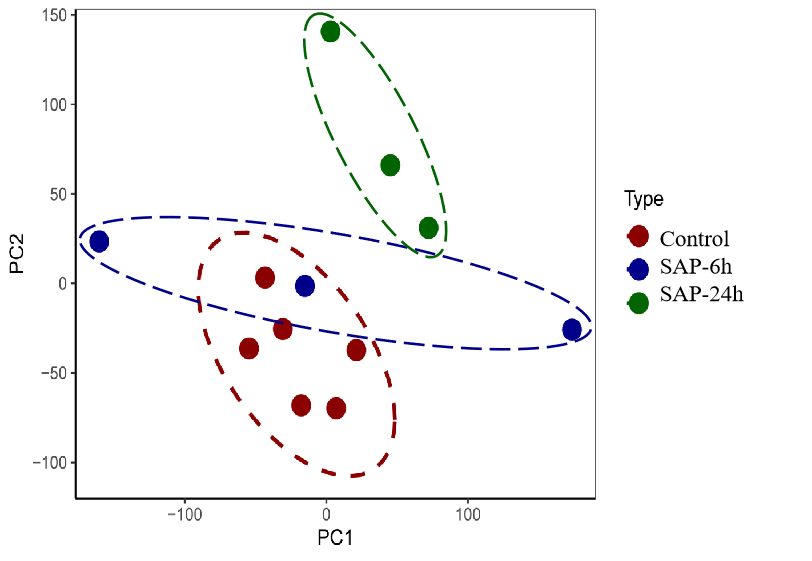
**

**Fig. S1.** Principal component analysis of SAP-6 h and SAP-24 h sub-subjects and normal controls. Dark red dots represent normal controls (*n* = 6), dark blur dots represent SAP-6 h AP mice (*n* = 3), and dark green dots represent SAP-24 h AP mice (*n* = 3).


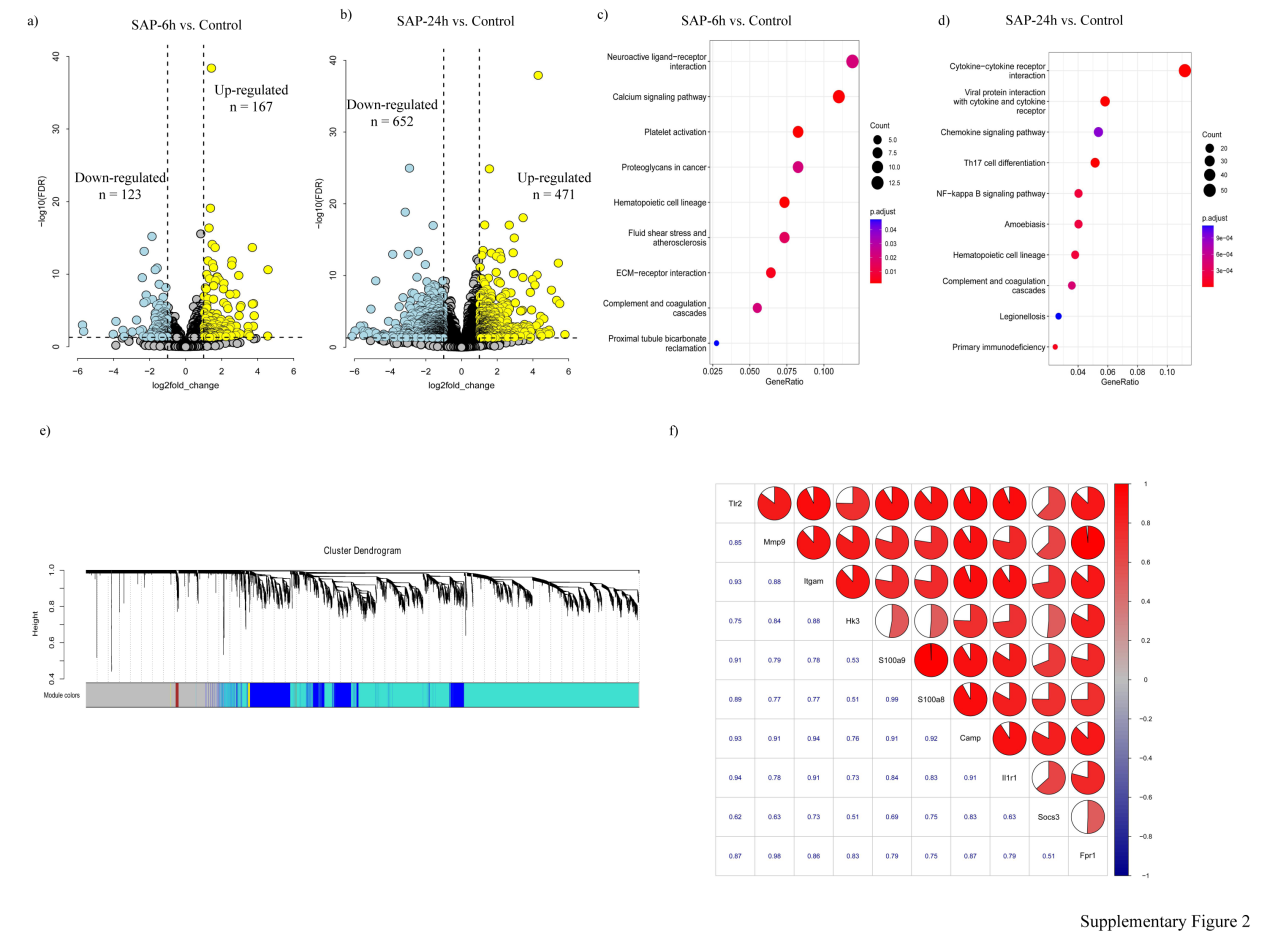


**Fig. S2.** Transcriptional characteristics in human and mice model. (a, b) Volcano plots of the mRNA expression profile of SAP 6 h *vs.* controls and SAP 24 h *vs.* controls in the AP animal model. Plotted along the x-axis is the mean log2 fold-change, and plotted along the y-axis is the negative logarithm of the log2 *P* values. The horizontal line represents the threshold for significant *P* values. Light yellow dots represent significantly upregulated DEGs, and light blue dots represent significantly downregulated DEGs. (c, d) Gene set enrichment analysis of DEGs for SAP 6 h *vs.* controls and SAP 24 h *vs.* controls in the AP experimental model. (e) Topological overlap matrix plots for AP modules. (f) Correlation matrix of the expression profiles of hub genes in the AP animal model.


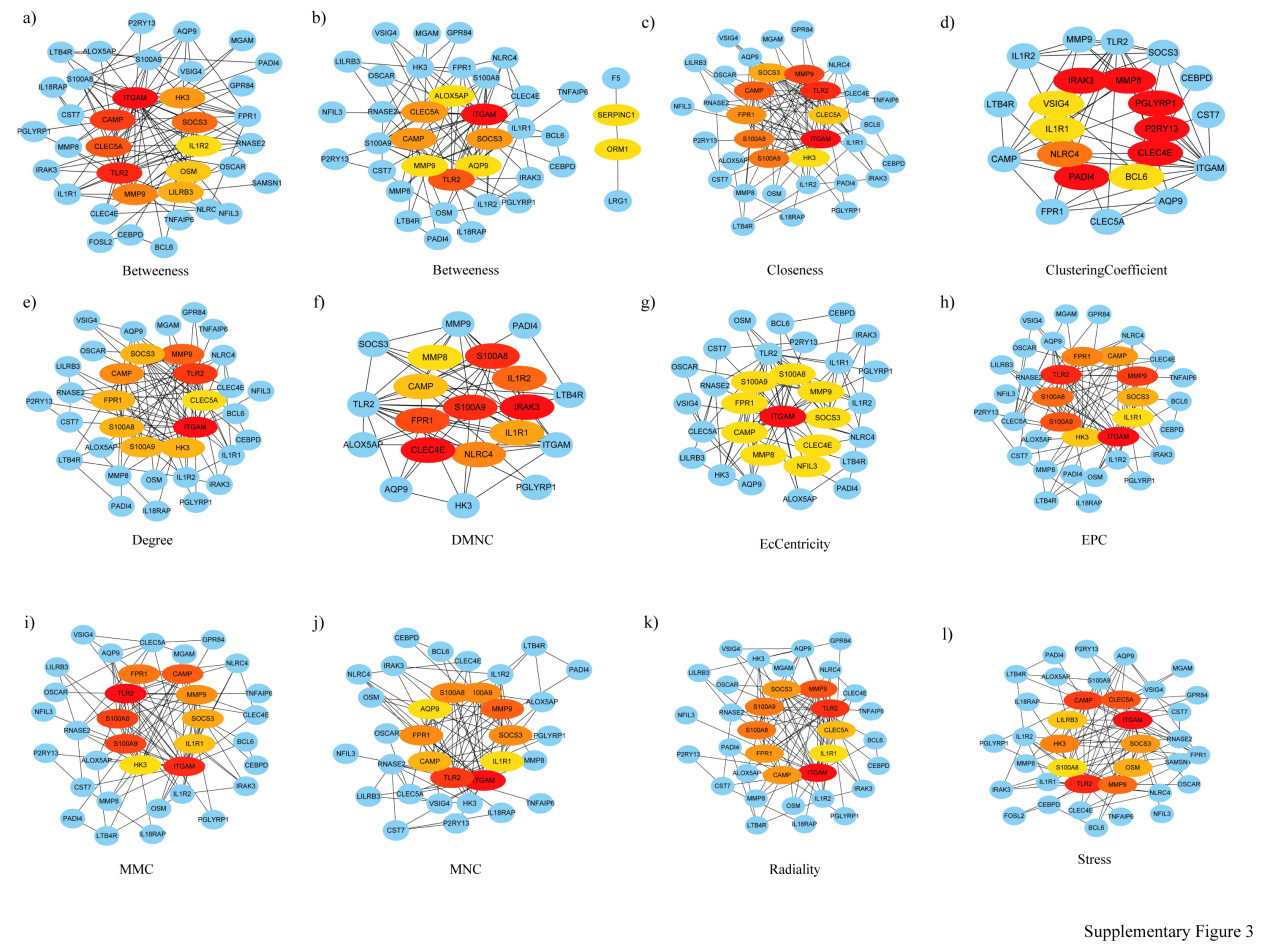


Fig. S3. Schematic diagram of highly interacted hub genes of AP using Cytohubba software with 12 different algorithms.


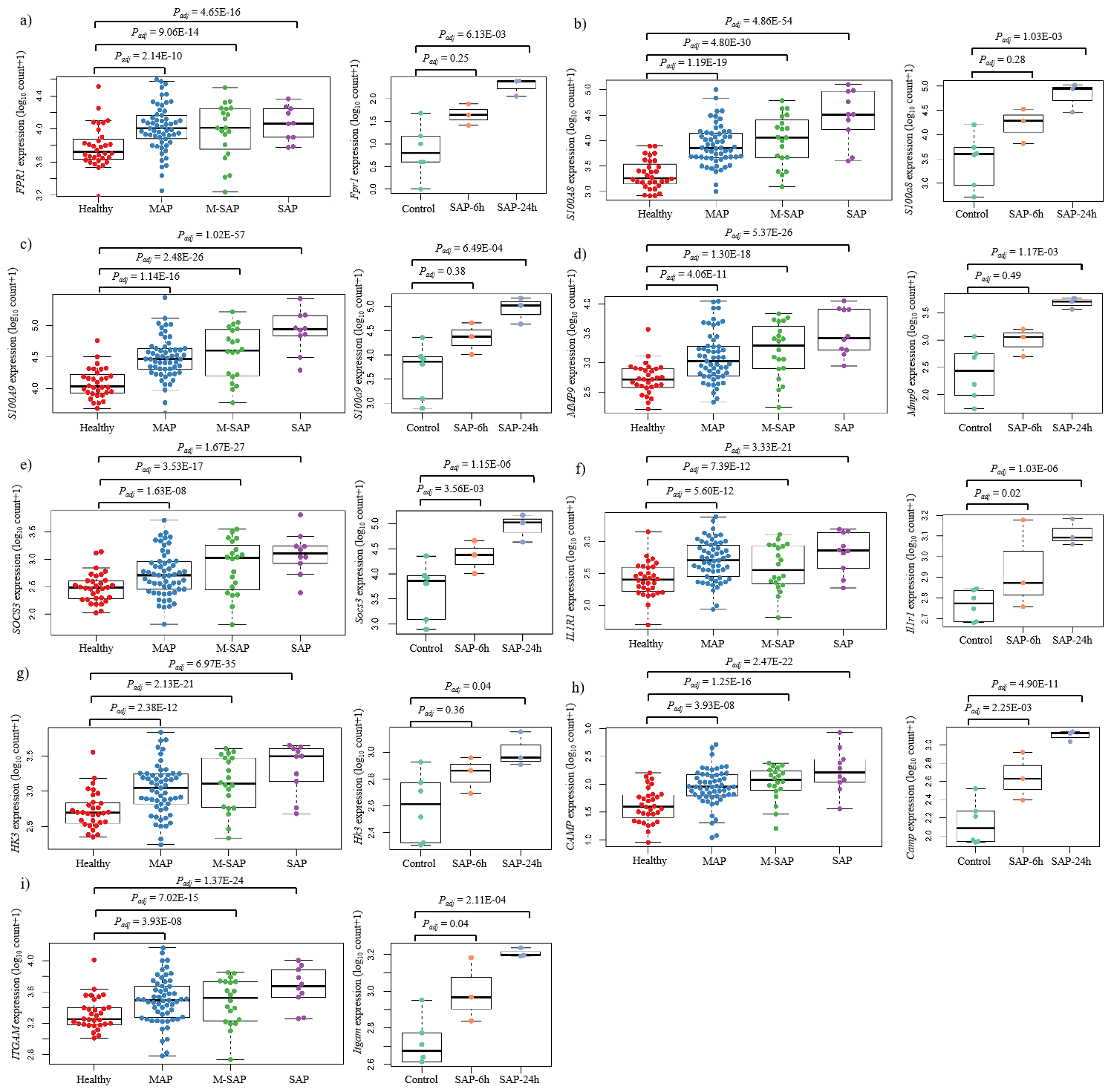


**Fig. S4.** Schematic diagram of identified epigenetic aberrations of hub genes of AP.


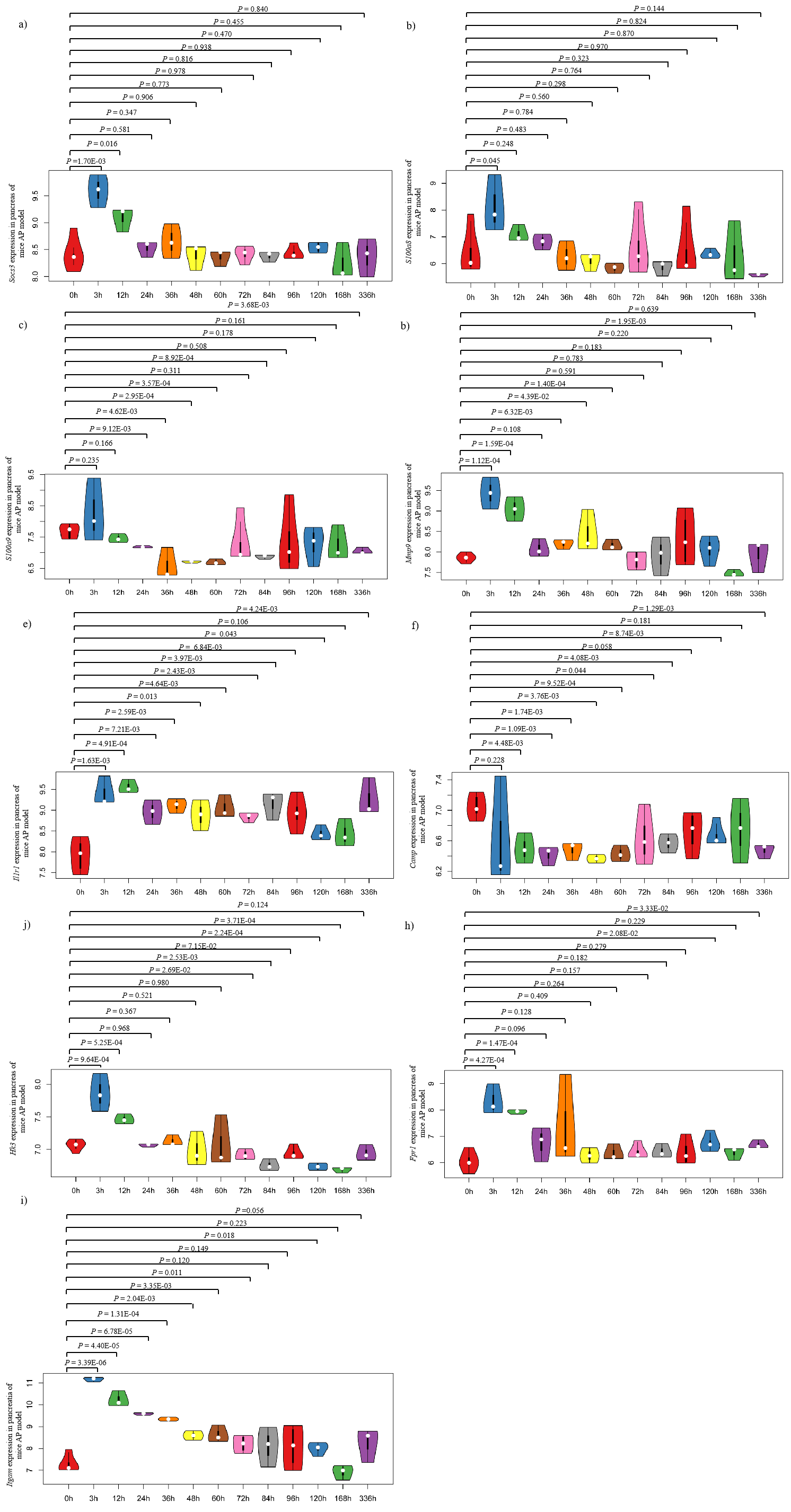


**Fig. S5.** The violin plot indicates dynamic changes in AP-related hub genes at thirteen consecutive time points in pancreatic tissue from the AP mouse model.
